# First record of winter pregnant males of two pipefish species in a Mediterranean coastal lagoon

**DOI:** 10.1111/jfb.70195

**Published:** 2025-08-22

**Authors:** Adrián Guerrero‐Gómez, Antonio Zamora‐López, Antonio Andrés Herrero‐Reyes, Francisco J. Oliva‐Paterna, Jorge Madrid‐Ruiz, Víctor M. Álvarez‐Navarro, Rocío Peñalver, Mar Torralva

**Affiliations:** ^1^ Department of Zoology and Physical Anthropology Faculty of Biology, University of Murcia Spain

**Keywords:** global warming, reproductive phenology, Syngnathidae breeding season, coastal lagoon

## Abstract

*Syngnathus abaster* Risso, 1827 and *Syngnathus typhle* L., 1758 are key components of fish assemblages in European transitional waters, with well‐documented reproductive cycles typically occurring from spring to autumn. However, recent warming trends in the western Mediterranean has raised questions about potential shifts in their breeding phenology. In January 2025, three pregnant *S. abaster* males and one *S. typhle* male were detected in the Mar Menor Coastal lagoon (western Mediterranean), representing the first recorded winter breeding event for both species. The size structure of *S. abaster* in winter 2025 also contrasted with colder winters, further supporting this interpretation. These findings may reflect a response to recent thermal anomalies and highlight the phenological plasticity of these species. To our knowledge, this is the first indication of potential phenological shifts in syngnathids from transitional waters, with implications for understanding how climate change may influence their reproductive dynamics.

Syngnathids are a charismatic flagship group with high relevance in the fish assemblages of European coastal lagoons (Pérez‐Ruzafa et al., 2007; Scapin et al., 2018; Vincent et al., 2011). Among them, *Syngnathus abaster* Risso, 1827 and *Syngnathus typhle* L., 1758 are the most representative species, exhibiting well‐documented reproductive strategies (Monteiro & Vieira, 2017; Rodríguez et al., 2021; Vincent et al., 1995). Males of both species carry fertilized eggs in fully enclosed brood pouches on their abdominal side, receiving multiple partial clutches from different females (Vincent et al., 1995). Mating success is size‐dependent, with larger males of *S. typhle* and *S. abaster* producing more offspring and also initiating breeding earlier in the season (Cunha et al., 2015; Flanagan et al., 2017). These species typically reproduce from spring to autumn as water temperatures rise, with no reports of winter reproduction (Flanagan et al., 2017; Franzoi et al., 1993; Monteiro & Vieira, 2017; Rodríguez et al., 2021; Tsikliras et al., 2010). However, recent episodic increases in water temperature in the western Mediterranean have raised questions about potential changes in syngnathid reproductive cycles (Atalah et al., 2024). Although there is currently no direct evidence linking temperature shifts to modifications in the breeding phenology of these species, changes in the reproductive cycles of other syngnathid species in offshore waters have been previously reported (Kirby et al., 2006; Monteiro et al., 2017), raising the question of whether warming trends may influence their reproductive cycles. Here, we present data on pregnant males of *S. abaster* and *S. typhle* collected during an 8‐year monitoring programme in the Mar Menor coastal lagoon. In the case of the more abundant species, *S. abaster*, we also examined winter size structure in relation to temperature patterns, aiming to explore whether recent thermal anomalies may be associated with changes in reproductive timing. These findings contribute to our understanding of syngnathid responses to environmental variability and provide relevant insights for the management of transitional ecosystems.

Between January 2018 and January 2025, we conducted seasonal fish sampling in the shallow areas of the Mar Menor using a beach seine net (depth <1.5 m, 24 sampling campaigns; see Zamora‐López et al., 2023). In addition, between October 2023 and January 2025, we have carried out seasonal fish sampling in the deeper areas (between 1.5 and 6.5 m) using an epibenthic trawl net (*n* = 6) known as a ‘gánguil’. After collection, fish specimens were temporarily placed in plastic containers and photographed on a millimetre‐scaled plastic tray, allowing for total body length measurements using ImageJ software (Schneider et al., 2012). To investigate phenological changes, we analysed the size distributions of the most abundant species, *S. abaster*, in shallow areas, which offered the greatest number of temporal replicates. *S. typhle* was excluded from size analyses due to very low winter catch rates (ranging between 0 and 6 individuals). Size distribution comparisons were initially conducted visually using histograms of relative frequency patterns between autumn and the subsequent winter. Differences in distributions were statistically tested using Kolmogorov–Smirnov tests for each autumn–winter pair. We then performed a permutational analysis of variance (PERMANOVA; Anderson, 2017) on winter size data, nesting years into two groups based on whether average water temperatures during the period 15 November to 15 December exceeded the species' thermal threshold for mating (18°C; Fedonenko et al., 2016; Silva, Monteiro, Vieira, & Almada, 2006). This period was selected because it represents the last part of the year in which temperatures may still exceed the mating threshold, and it precedes the winter sampling dates, falling within the reported duration of embryo development in the brood pouch for this species (1 month; Silva, Monteiro, Almada, & Vieira, 2006). Additionally, we examined mean size, size evenness, size diversity, log‐transformed size diversity and log‐transformed size dispersion, following the metrics described by Quintana et al. (2016). These variables were correlated with mean temperature during the same pre‐winter period using Spearman's rank correlation. All statistical analyses were conducted using R software (Version 4.3.0) (R Core Team, 2025), and temperature data for the Mar Menor were obtained from CARM (2025).

Here, we report the detection of three pregnant *S. abaster* males and one *S. typhle* male in the waters of the Mar Menor coastal lagoon (western Mediterranean Sea) between 14 and 21 January 2025 (Figure 1). An individual of *S. abaster* (85 mm total length) was detected in shallow areas, while the other two (60 mm and 106 mm) and the *S. typhle* individual (223 mm) were found in deeper areas. These species breed from spring to autumn, covering at most February to October for *S. abaster* and April to October for *S. typhle* (Monteiro & Vieira, 2017; Takahashi et al., 2003; Tsikliras et al., 2010). In both species, males brood their embryos in a brood pouch for approximately 1 month, although low temperatures may extend this period (Ahnesjö, 2008; Silva, Monteiro, Almada, & Vieira, 2006). Therefore, the pregnant individuals observed in January likely mated during November–December, when water temperatures remained within the suitable range for reproduction. This pattern was especially marked in 2024, when suitable conditions persisted until early December (Figure 2), suggesting that anomalously warm autumns may lead to an extension of the species' known breeding season.

**FIGURE 1:**
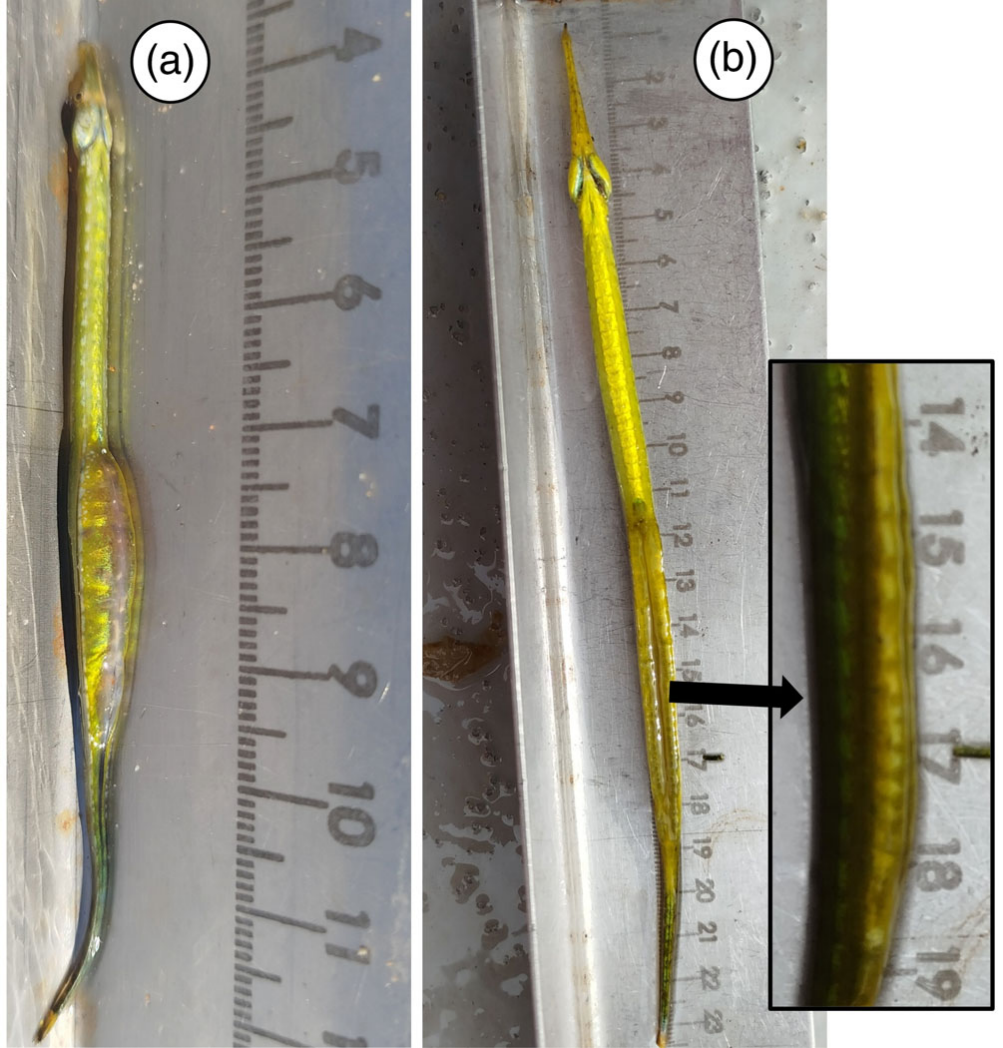
Pregnant males of *Syngnathus abaster* (a) and *Syngnathus typhle* (b) observed in January 2025 in Mar Menor coastal lagoon (western Mediterranean Sea).

**FIGURE 2:**
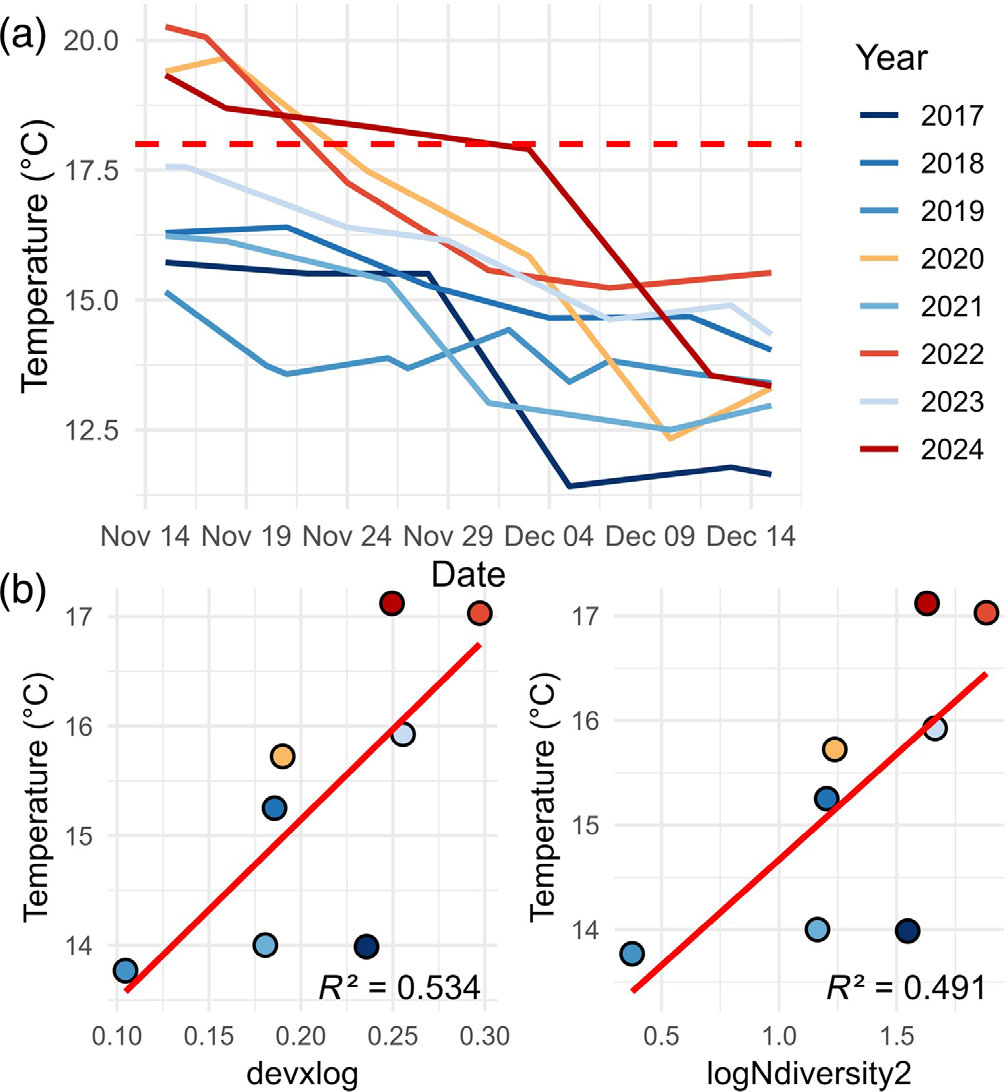
(a) Temporal evolution of water temperature for 15 November to 15 December by year in the Mar Menor (western Mediterranean Sea. The red dashed line represents the minimum temperature that allows *Syngnathus abaster* matings (18°C). (b) Linear regression between temperature mean (15 November to 15 December) by year and the significant correlated size metrics of *S. abaster* captured in winter in shallow areas of Mar Menor (western Mediterranean Sea).

The size distribution of *S. abaster* in winter 2025 more closely resembled that of the preceding autumn, as indicated by the highest *p* value and a low *D* statistic in the Kolmogorov–Smirnov tests performed between seasonal pairs (Figure 3). Additionally, 2025 exhibited a unique transition in size structure between autumn and winter, characterized by a decrease in mean size and an increase in diversity and evenness, driven by the emergence of new juveniles. Other years with warm late autumns, such as 2022–2023, partially mirrored this pattern but did not show a reduction in mean size. This shift aligns with the unusually warm conditions recorded in late autumn 2024, which provided approximately 10 more days of suitable temperatures for *S. abaster* mating compared to previous warm years (2020–2021 and 2022–2023) (Figure 2). Furthermore, this pattern was supported by the PERMANOVA test, which revealed that size structure was significantly influenced by warmer years—those exceeding 18 °C after mid‐November—highlighting the potential role of elevated water temperatures during this period in triggering phenological changes in syngnathid species (Table 1). Moreover, average water temperature during these dates showed significant positive correlations with diversity and size deviation descriptors of the size structure, but only after applying a logarithmic transformation (Table 2 and Figure 2). This suggests that higher temperatures promote greater variability among smaller (juvenile) size classes. The log transformation dampens the influence of the largest individuals, highlighting changes within the juvenile spectrum and further supporting an extended breeding period for the species under such conditions.

**FIGURE 3:**
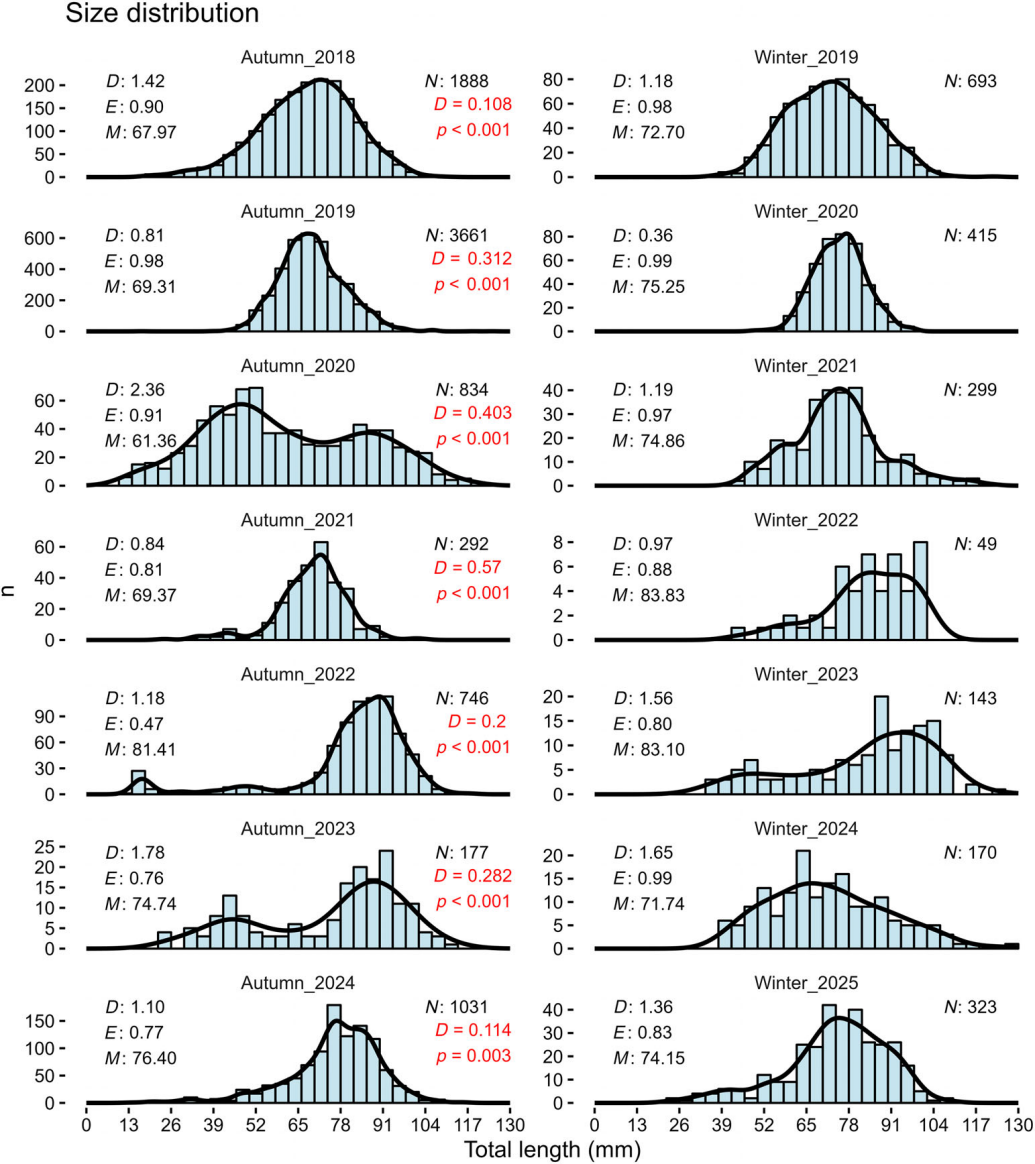
Size distribution of *Syngnathus abaster* captured in autumn (October) and winter (January) from 2018 to 2024 in the shallow areas of the Mar Menor coastal lagoon (western Mediterranean Sea. Total number of catches (*N*), size diversity (*D*), size evenness (*E*) and size mean (*M*). The red letters indicate the Kolmogórov–Smirnov test results between autumn and the following winter (*D* statistic and *p* value).

**TABLE 1:** Parameters resulted from the one factor PERMANOVA test based on the Euclidean distances of *S. abaster* winter sizes in the shallow areas of Mar Menor coastal lagoon (western Mediterranean Sea).

	*df*	MS	*R* ^2^	*F*	*p*
T_group	1	6245.628	0.011	30.357	<0.001***
Year(T_group)	6	22,396.054	0.040	18.143	<0.001***
Residual	2536	521,752.178	0.948		
Total	2543	550,393.86	1		

*Note*: T_group was defined according to the occurrence of temperatures suitable for *S. abaster* reproduction (18°C) between 15 November and 15 December of the assessed years. Year was nested in this factor. Mean squares (MS), *F* values (*F*), coefficient of determination (*R* ^2^) and degrees of freedom (*df*). Significance levels (*p*): *p < 0.05, **p < 0.01, ***p < 0.001.

**TABLE 2:** Spearman correlation matrix of mean temperature between 15 November and 15 December, and size distribution metrics of *S. abaster* captured in winter in shallow areas of Mar Menor coastal lagoon (western Mediterranean Sea).

Variable	Correlation coefficient	*p*
diversity2	0.619	0.115
evenness	−0.548	0.171
logNdiversity2	0.786	0.028*
devxlog	0.786	0.028*
meanx	0	1

On the other hand, between late 2019 and 2021, the Mar Menor coastal lagoon experienced successive critical eutrophic episodes, resulting in severe and widespread mass mortality events affecting aquatic fauna (Fernández‐Alías et al., 2022). These disturbances led to a marked decline in the abundance of *S. abaster* during the subsequent years (2021–2024; Guerrero‐Gómez et al., 2024; Zamora‐López et al., 2023), as evidenced by notably reduced detection rates and a capture frequency approximately 50% lower than in 2025 (Figure 3). The absence of brooding males during the winter of 2020–2021 and 2022–2023, despite thermal conditions relatively comparable to those in 2024–2025, may reflect diminished population densities post‐mortality, potentially limiting mating opportunities and reducing the probability of encounter. Moreover, these eutrophic stress events may have compromised the somatic condition of surviving individuals, thus could have diminished their reproductive readiness or capacity (Almeida et al., 2023).

The influence of temperature on syngnathid reproductive phenology is well documented, with numerous studies linking monthly increased temperatures to breeding activity (Monteiro et al., 2005; Silva et al., 2007; Silva, Monteiro, Vieira, & Almada, 2006; Taylan et al., 2018). There is also evidence suggesting that global warming is advancing and extending the breeding season of some offshore syngnathids, potentially altering sexual selection dynamics (Kirby et al., 2006; Monteiro et al., 2017). In addition, climate projections indicate a potential decline in Mediterranean syngnathid populations under continued warming scenarios (Monteiro et al., 2023). In line with this, laboratory experiments have also confirmed the potential thermal stress induced by climate change on syngnathid species (Costa et al., 2023). However, to our knowledge, this is the first documented evidence of phenological shifts in syngnathids from transitional waters associated with an unusual increase in temperature. These situations could become more frequent under ongoing global warming (Atalah et al., 2024). Confined transitional systems like the Mar Menor are particularly vulnerable to climate change (De Pascalis et al., 2012), making our observations highly relevant for understanding the potential response of this charismatic group in European transitional ecosystems. Further research should assess the hatching success and survival of juveniles from winter matings to better infer the long‐term implications of climate‐induced shifts in reproductive timing.

## AUTHOR CONTRIBUTIONS

**Adrián Guerrero‐Gómez:** Writing – original draft, methodology, formal analysis, data curation, conceptualization. **Antonio Zamora‐López:** Writing – review and editing, methodology, investigation. **Antonio Andrés Herrero‐Reyes:** Writing – review and editing, methodology, investigation. **Francisco José Oliva‐Paterna:** Writing – review and editing, validation, supervision, project administration, investigation, funding acquisition, conceptualization. **Jorge Madrid‐Ruiz:** Methodology, investigation. **Víctor Manuel Álvarez‐Navarro:** Methodology, investigation. **Rocío Peñalver:** Methodology, investigation. **Mar Torralva:** Writing – review and editing, validation, supervision, project administration, methodology, investigation, funding acquisition, conceptualization.
